# Suicide Gene Therapy Mediated with Exosomes Produced by Mesenchymal Stem/Stromal Cells Stably Transduced with HSV Thymidine Kinase

**DOI:** 10.3390/cancers12051096

**Published:** 2020-04-28

**Authors:** Andrea Pastorakova, Jana Jakubechova, Ursula Altanerova, Cestmir Altaner

**Affiliations:** 1 Institute of Medical Biology, Genetics and Clinical Genetics, Faculty of Medicine, Comenius University in Bratislava, Sasinkova 4, 811 08 Bratislava, Slovakia; 2 Stem Cell Preparation Laboratory, St. Elisabeth Cancer Institute, Heydukova 10, 812 50 Bratislava, Slovakia; 3 Biomedical Center, Cancer Research Institute, Slovak Academy of Sciences, Dubravska cesta 9, 845 05 Bratislava, Slovakia

**Keywords:** *HSVTK*, GCV, MSCs, suicide gene *HSVTK* exosomes

## Abstract

Mesenchymal stem/stromal cells (MSCs) prepared from various human tissues were stably transduced with the suicide gene herpes simplex virus thymidine kinase (*HSVTK)* by means of retrovirus infection. *HSVTK*-transduced MSCs express the suicide gene and in prodrug ganciclovir (GCV) presence induced cell death by intracellular conversion of GCV to GCV-triphosphate. The homogenous population of *HSVTK*-MSCs were found to release exosomes having mRNA of the suicide gene in their cargo. The exosomes were easily internalized by the tumor cells and the presence of ganciclovir caused their death in a dose-dependent manner. Efficient tumor cell killing of glioma cell lines and primary human glioblastoma cells mediated by *HSVTK*-MSC exosomes is reported. Exosomes produced by suicide gene transduced MSCs represent a new class of highly selective tumor cell targeted drug acting intracellular with curative potential.

## 1. Introduction

The natural behavior of mesenchymal stem cells (MSCs) toward the target tumor has led to the idea of using them for the delivery of anti-cancer compounds into tumors [1]. MSC tumor tropism inspired us to develop two suicide gene directed enzyme prodrug therapy systems mediated by MSCs [2]. The *yCD:UPRT*-MSC/5-FC system is based on MSCs engineered to express fused yeast cytosine deaminase::uracil phosphoribosyl transferase (*yCD::UPRT*) with 5-fluorocytosine (5-FC) as the prodrug [3]. We have shown that the yCD:UPRT-MSC/5-FC system effectively inhibited the growth of human colon carcinoma [3], melanoma, [4], and prostate carcinoma [5,6] in nude mice in preclinical studies. Adipose tissue derived MSCs (AT-MSCs) engineered to express the *yCD::UPRT* gene were shown to induce curative therapeutic effect in a substantial number of rats with intracranial glioblastoma in a preclinical model [7,8,9].

The field of suicide gene therapy was pioneered by studies where thymidine kinase of herpes simplex virus (*HSVTK*) was stably introduced to tumor cells as a way of controlling the chemosensitivity of tumor cells [10]. The second suicide gene directed enzyme prodrug therapy system we established was the *HSVTK*-MSC/GCV system [11]. This system used ganciclovir (GCV) as the prodrug in AT-MSCs engineered to express the *HSVTK* gene from DNA retroviral provirus integrated in cellular DNA. The retrovirus vector used for MSC-transduction enables a homogenous cell population of transduced cells to be obtained by G418 antibiotic selection [12]. Since then, the suicide gene therapy has expanded to several suicide gene systems for cancer therapy [13].

We have recently reported that MSCs derived from various human tissue types that had been engineered to express *yCD::UPRT* gene release exosomes possessing mRNA of the *yCD::UPRT* suicide gene in their cargo. We named them MSC suicide gene exosomes. When these exosomes were applied to tumor cells, the exosomes were internalized by the recipient tumor cells and in 5-FC presence, the prodrug effectively triggered dose-dependent tumor cell death. The tumor cell death was caused by endocytosed exosomes via an intracellular conversion of the prodrug 5-FC to 5-fluorouracil (5-FU) by cytosine deaminase. In addition, an UPRT part of the enzyme catalyzes the direct conversion of 5-FU to 5-fluorouridinemonophosphate (5-FUMP), an irreversible inhibitor of DNA synthesis [14].

Here, we report on the analysis of the *HSVTK*-MSC/GCV system with regard to the release of extracellular vesicles of the suicide gene exosome type. MSCs engineered to express thymidine kinase of herpes simplex virus were found to produce exosomes possessing mRNA of the *HSVTK* suicide gene in their cargo, similar to that as the *yCD:UPRT*-MSC/5-FC system. The *HSVTK*-MSC suicide gene exosomes upon tumor cell internalization in the presence of prodrug ganciclovir induced cell death by intracellular conversion of GCV to GCV-triphosphate (GCV-TP) in a dose-dependent manner.

## 2. Results

### 2.1. A Schematic Workflow of All Steps Performed in This Study

**Figure 1:**
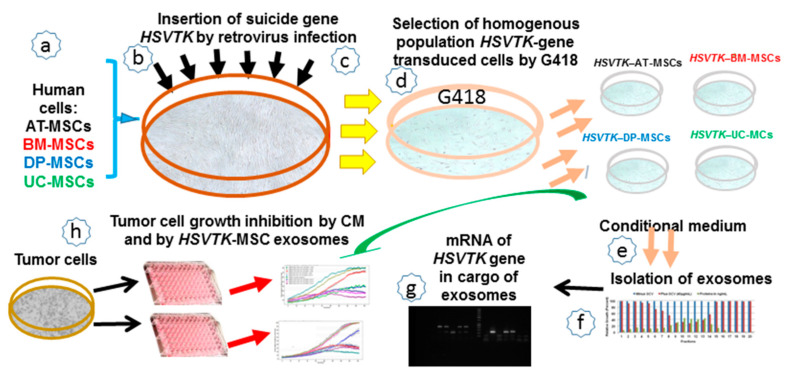
Steps taken during the experiment. (**a**,**b**) Isolation and expansion of MSCs from various tissues; (**c**) Infection of MSCs with retrovirus carrying *HSVTK* suicide gene; (**d**) Selection of cell population of suicide gene-transduced cells; (**e**) Harvesting of conditional medium; (**f**) Isolation of exosomes from conditional medium (CM) by size-exclusion chromatography; (**g**) Detection of mRNA of *HSVTK* suicide gene in the cargo of exosomes; (**h**) Tumor cell growth inhibition with CM and *HSVTK*-exosomes. Abbreviations: MSCs, mesenchymal stem cells; DP-MSCs, dental pulp MSCs; AT-MSCs, adipose-tissue MSCs; BM-MSCs, bone marrow MSCs; UC-MSCs, umbilical cord MSCs; *HSVTK*, thymidine kinase of herpes simplex virus gene.

MSCs prepared from various human tissues were engineered to express the suicide gene *HSVTK. HSVTK*-transduced MSCs secrete exosomes carrying mRNA of the suicide gene in their cargo. The exosomes internalized by the recipient tumor cells in GCV presence caused their death in a dose-dependent manner (Figure 1).

### 2.2. MSCs Obtained from Various Human Tissues Can Be Stably Transduced with the HSVTK Suicide Gene

**Figure 2:**
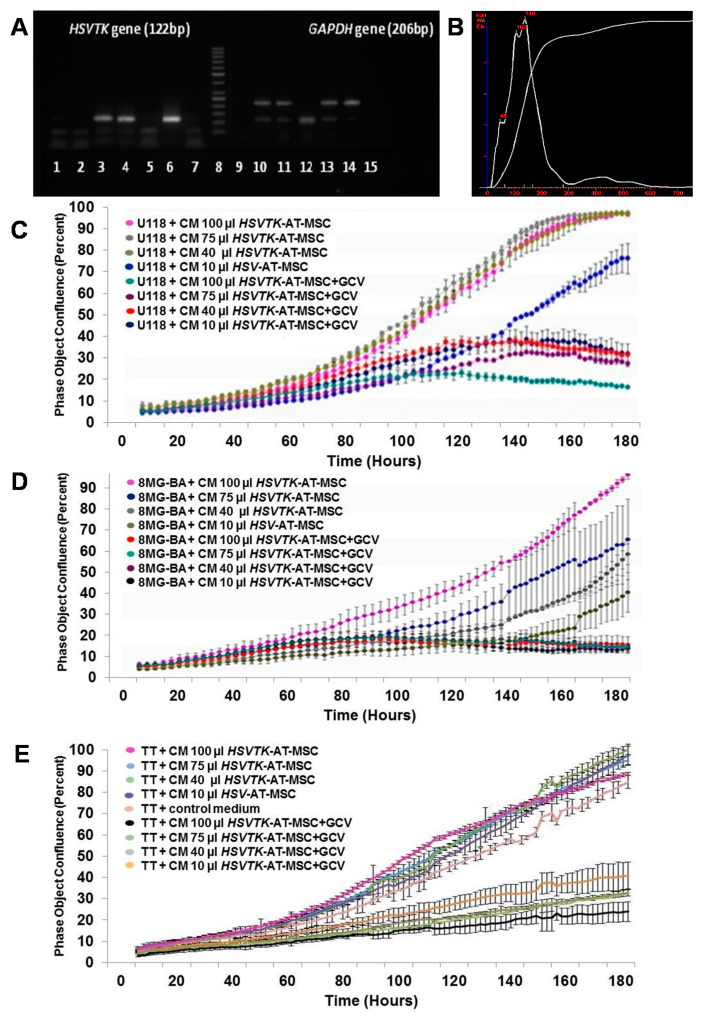
Expression of the *HSVTK* gene in homogenous cultured *HSVTK*-MSCs and assessment of growth inhibition activity of various tumor cells by CM from MSCs engineered to express the *HSVTK* gene. (**A**) Detection of HSVTK sequence presence in DNA of MSC cells after polymerase chain reaction (PCR) was visualized by 2% agarose gel electrophoresis: HSVTK PCR gel (1–7): (1 and 7) reaction mixture with no DNA (NTC); (2) PC3 cell (negative control); (3) *HSVTK*-AT-MSCs; (4) GP-AM/TK; (5) AT-MSCs; (6) plasmid pAPtk (positive control); (8) 50 bp ladder; *GAPDH* PCR gel (9–15): (9 and 15) reaction mixture with no DNA (NTC); (10) PC3 cell (positive control); (11) *HSVTK*-AT-MSCs; (12) GP-AM/TK (negative control); (13) AT-MSCs; (14) human genomic DNA (positive control). (**B**) Detection of exosomes released from *HSVTK* gene transduced MSCs by Nanosight. (**C**) Growth curve of human glioma tumor cell line U118 treated with CM of *HSVTK*-AT-MSCs in the presence and absence of GCV. Killing activity of CM from *HSVTK*-AT-MSCs was dose-dependent. (**D**) Dose dependence of *HSVTK*-AT-MSC conditional medium growth inhibition activity tested on human glioma tumor cell line 8MG-BA. Glioblastoma cell line 8MG-BA was found to be more sensitive to the *HSVTK* killing effect (Figure 2D). (**E**) CM of *HSVTK*-AT-MSCs was killing human slowly growing medullary carcinoma cells TT resistant to radiotherapy in a dose-dependent manner in GCV presence and absence. Starting number of tumor cells of all three tumor cell lines in the well of 96-well plate was 3 × 10^4^ at the beginning of the treatment.

We prepared MSCs from various human tissues and transduced them with the *HSVTK* gene by means of retrovirus infection. The design of the retrovirus vector used in this study, being a bicistronic construct with the *HSVTK* gene separated by IRES from the *NEO* gene, allowed for the selection of the homogenous population of *HSVTK*-transduced cells with the G418 antibiotic. All four MSCs isolated from different tissue origins transduced with *HSVTK* (*HSVTK*-MSCs) and selected for their transgene presence were found to have suicide genes integrated into their cell DNA as DNA provirus (Figure 2A).

### 2.3. The Homogenous Population of Mesenchymal Stem Cells (MSCs) Transduced with Herpes Simplex Virus Thymidine Kinase (HSVTK) Gene Release Exosomes

The conditional medium (CM) prepared from MSCs and *HSVTK*-MSCs was analyzed for the presence of extracellular vesicles. Nanoparticle tracking analysis of CM filtered through 0.20 mm filters detected round exosomes in the range of 37 to 135 nm. The concentration of exosomes in all tested CM fluctuated at around 8.75 × 10^9^ particles/mL, regardless of naϊve or HSVTK-MSCs (Figure 2B).

### 2.4. HSVTK-MSCs and Corresponding Conditional Medium (CM) Induce Tumor Cell Death in the Prodrug Ganciclovir Presence in a Dose-Dependent Manner

Human tumor cell growth was monitored in 96 microplates using the real-time live-cell monitoring IncuCyte system and controlled by the MTT test (Promega Corporation, Madison, WI 53711 USA).

The testing system for CM from *HSVTK*-MSCs enabled us to distinguish between growth support induced by CM (GCV not added to CM) and tumor cell growth inhibiting activity (GCV added to CM). CM of naïve MSCs and *HSVTK*-MSCs supported growth of recipient tumor cells similarly in GCV absence, but a strong growth inhibition of tumor cells was found when GCV was added to CM of *HSVTK*-MSCs. The efficacy of *HSVTK*-MSC CM to inhibit the growth of several tumor cell lines was tested. CM of *HSVTK*-MSCs with GCV inhibited the growth of human glioma tumor cell line U118 (Figure 2C) and glioma 8MG-BA (Figure 2D) in a dose-dependent manner. Slow growing medullary thyroid carcinoma cells TT, known for their resistance to radio- and chemotherapy, were also effectively inhibited in their growth by *HSVTK*-MSC CM/GCV treatment (Figure 2E).

### 2.5. The Efficiency of HSVTK-AT-MSCs Compared to Matched CM in the Growth Inhibiting Activity of Glioma Cell Lines and Primary Glioblastoma Cells

**Figure 3:**
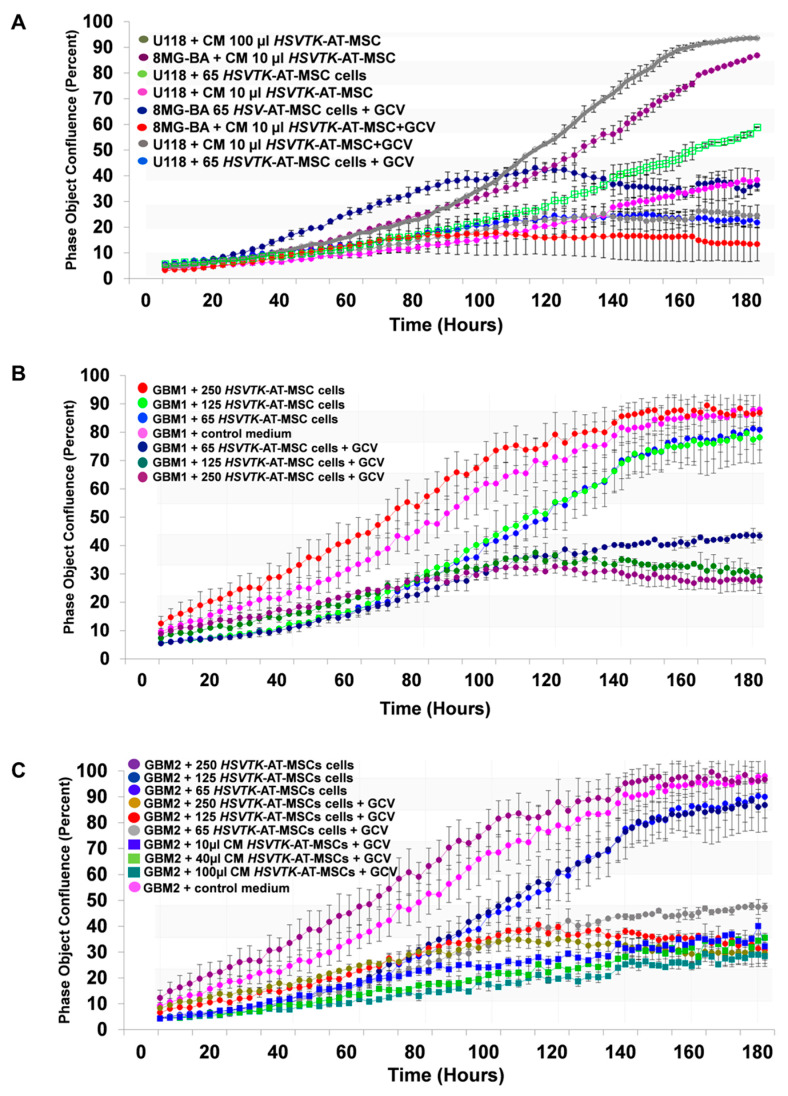
Comparison of tumor cell killing activity of *HSVTK*-AT-MSC cells versus corresponding CM on glioma cell lines and on primary glioblastoma cells**.** (**A**) Assessment of growth inhibition activity of *HSVTK*-AT-MSC-CM and *HSVTK*-AT-MSC cells compared on human glioma cell lines U118 and 8MG-BA. Starting number of tumor cells in the well of 96-well plate was 3 × 10^4^. (**B**) Evaluation of growth inhibition of human primary glioblastoma cells GBM1 by *HSVTK*-MSC cells. (**C**) Killing of human primary glioblastoma cells GBM2 by *HSVTK*-MSC cells and corresponding CM.

*HSVTK*-AT-MSC cells as well as corresponding CM co-cultivated with glioma cells U118 and 8MG-BA in the presence of prodrug GCV inhibited their growth in a dose-dependent fashion (Figure 3A). Then comparison revealed that 65 cells of *HSVTK*-MSCs possessed the same killing activity as 10 µL of CM (24 h harvest from 10^6^ cells). Primary human glioblastoma GBM1 and GBM2 cultivated as 2D cells were similarly tested in the independent experiment. *HSVTK*-AT-MSC cells inhibited the growth of GBM1 in a dose-dependent manner. In six days, 65 therapeutic cells reduced 10^4^ starting tumor cells to more than half (Figure 3B). Comparison of the tumor killing efficiency of *HSVTK*-AT-MSCs CM versus *HSVTK*-AT-MSC cells tested on primary glioblastoma cells GBM2 revealed a slightly higher efficiency of CM over cells (Figure 3C). *HSVTK*-MSC CM in the absence of GCV stimulated growth of both the primary glioma cells tested, but the addition of GCV killed them both completely in a dose dependent fashion.

### 2.6. Exosomes Are Responsible for the Tumor Cell Killing Activity in HSVTK-MSC CM

**Figure 4:**
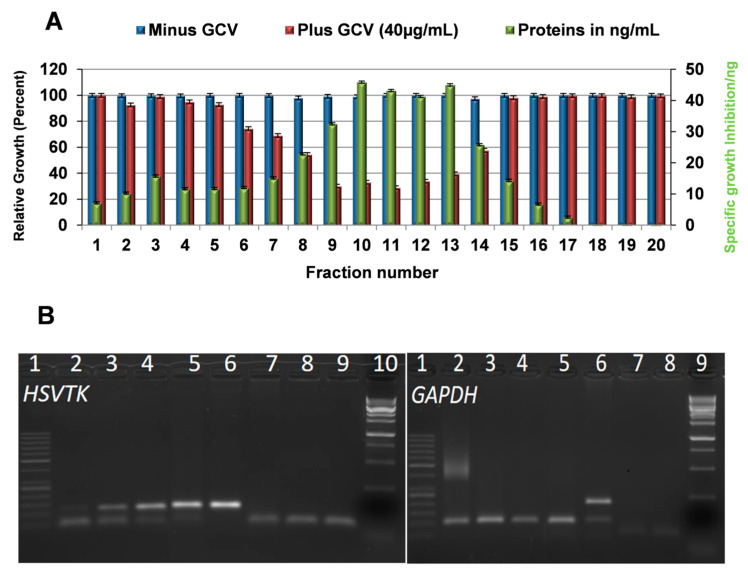
Assessment of glioblastoma cell growth inhibition activity by exosomes with mRNA of *HSVTK* in their cargo. (**A**) Elution profile of CM fractionated by the size-exclusion chromatography in a Sepharose CL-4B column. Each fraction was tested for tumor cell growth (absence GCV) and tumor cell growth inhibition (presence GCV). Relative growth inhibition to protein presence was calculated. (**B**) RNA isolated from exosomes of the *HSVTK* gene transduced UC-MSCs was reverse transcribed (RT) and *HSVTK* and *GAPDH* cDNA was PCR amplified. Presence of mRNA of *HSVTK* gene specific transcripts was visualized by 2% agarose gel electrophoresis. *HSVTK*-RT-PCR gel: (1) O’RangeRuler 50 bp DNA Ladder; (2) exosomal RNA of MSC (RT with oligo (dT)_18_primer_s_); (3) exosomal RNA of UC-MSC (RT using random hexamers); (4) total RNA *HSVTK*-MSCs (mRNA *HSVTK* positive control); (5) total RNA *HSVTK*-melanom cells (mRNA *HSVTK* positive control); (6) plasmid pAPtk (PCR *HSVTK* positive control); (7) human genome DNA (PCR negative control); (8) RT reaction mixture with no RNA in reverse transcription (NTC); (9) PCR reaction mixture with no DNA in mixture (NTC); (10) 1 kb DNA ladder Solis; *GAPDH* RT-PCR gel: (1) O’RangeRuler 50 bp DNA Ladder; (2) exosomal RNA of MSC (RT with oligo (dT)_18_primer_s_); (3) exosomal RNA of MSC (RT using random hexamers); (4) total RNA *HSVTK*-MSCs (mRNA *HSVTK* positive control); (5) total *RNA HSVTK*-uveal melanoma cells *(mRNA HSVTK* positive control); (6) mixture of genome DNA and cDNA of *HSVTK*-MSCs (RT-PCR and PCR positive control); (7) RT reaction mixture with no RNA in reverse transcription (NTC); (8) PCR reaction mixture with no DNA in mixture (NTC); (9) 1 kb DNA ladder Solis.

The MSC secretome, represented by CM and CM from the corresponding *HSVTK*-AT-MSCs, is composed of plenty of biologically active low molecular weight factors such as cytokines, chemokines, and growth factors together with extracellular vesicles. To find out whether exosomes or biological factors in the CM of *HSVTK*-AT-MSC are responsible for the tumor cell growth inhibiting activity, we analyzed the CM by size-exclusion chromatography on a Sepharose CL-4B column. All obtained fractions were tested for their tumor killing activity in GCV presence/absence concerning U118 tumor cell growth activity. Figure 4A shows that the most of the tumor growth inhibiting activity was localized to the nanoparticle fractions, where the highest protein level was detected. In addition, the highest number of nanoparticles was found in the same fractions by means of NanoSight analysis. MSCs transduced with the *HSVTK* gene secreted a heterologous population of exosomes with regard to the size. From the several methods for their isolation we tested, the size exclusion chromatography on the Sepharose gels was found to cover all biologically active particles. The existence of small biologically active nanoparticles [15] that did not sediment, even at the extreme conditions of ultracentrifugation, led us to prefer CM over isolated exosomes for the assessment of the tumor cell killing activity.

### 2.7. Messenger Ribonucleic Acid (mRNA) of the Prodrug Converting Gene Is Present in the Cargo of Exosomes Released from HSVTK-MSCs

Total RNA isolated from exosomes was subjected to PCR amplification using primers for *HSVTK* and *GAPDH*. RT-PCR analysis using random hexamer primers revealed the presence of mRNA specific to the *HSVTK* gene of the *HSVTK*-MSCs analyzed (Figure 4B). Therefore, the *HSVTK*-transduced MSCs packed the suicide gene *HSVTK* mRNA into the exosome’s cargo. The internalization of exosomes into tumor cells and subsequent intracellular translation of m-RNA to an enzyme converting GCV to GCV-TPH was responsible for tumor cell death. Real time live tumor cell monitoring, treated with CM in GCV presence, revealed that the phosphorylation of GCV and incorporation into DNA of recipient tumor cells took approximately 36–40 h.

### 2.8. Tumor Cell Growth Inhibition Efficacy of Exosomes from HSVTK Gene Transduced MSCs of Various Tissues Origin

The efficiency of therapeutic exosomes derived from various types of MSCs differed in triggering tumor cell death. The *HSVTK*-gene transduced MSCs of different tissue origin were prepared by retrovirus infection, yielding cells with random integrated DNA proviruses. The presence of the *NEO* gene linked to the *HSVTK* gene by IRES in the retrovirus vector allowed for G418 antibiotic selection of the homogenous population of transgene containing cells. We had asked whether the transgene copy number in various MSCs differed by means of qRT-PCR. We found different levels of transgene integration in the cellular DNA. The level of *HSVTK* gene expression in most tested cells concurred with the copy number of the integrated gene and was also reflected in tumor cell death ability.

## 3. Discussion

Recently we have reported the release of suicide gene exosomes from MSCs with the stably integrated suicide gene *yCD::UPRT*. Tumor cell targeting of MSC suicide gene exosomes enables the conversion of nontoxic 5–FC to a widely used cytostatic compound 5-FU intracellular. MSC suicide gene-exosome treatment represents a novel type of cell-free cancer gene therapy with higher efficiency and safety standards [14]. We had extended our studies to a second suicide gene system *HSVTK*-MSC/GCV, which we had developed years ago [11]. Here, we report evidence that the H*SVTK* gene integrated in MSC DNA under strong retrovirus promoter expresses mRNA of the transgene and packs it into the cargo of releasing exosomes.

The neural tropism of HSV predetermines the *HSVTK* gene to be used for the treatment of brain tumors, being the most commonly used in preclinical and clinical studies against glioma [16,17,18]. This enzyme has a high affinity to the guanine analog ganciclovir, which is further phosphorylated by endogenous cellular enzymes. During DNA synthesis, tri-phosphorylated GCV is incorporated into the DNA strand, blocking chain elongation and leading to cell death. It has been repeatedly proven that the *HSVT*K/GCV treatment results in the death not only of recipient cells, but also of the surrounding non-recipient tumor cells. This phenomenon is known as the bystander effect and involves the transference of toxic phosphorylated GCV by gap junctions [11,19,20,21]. *HSVTK*-MSCs releasing exosomes with mRNA of the suicide gene in the exosome’s cargo when applied to glioma cells increase tumor cell killing activity. Thus, the efficacy of prodrug gene therapy for cancer mediated by the *HSVT*K-MSCs/GCV system is manifested not only through the bystander effect, but mainly through exosomes that internalize tumor cells. Human glioblastoma is a heterologous tumor composed of differentiated tumor cells and a small portion of tumor-initiating cells, glioma stem cells (GSC), which have a high tumorigenic potential and low proliferation rate. The aggressive nature of GBM, manifested by its incurability, is mainly due to GSC [22]. It has been reported that *HSVTK* suicide gene therapy was less efficient in GSC than in tumor cells because of the elimination of GCV by ABCG2-mediated efflux [23]. The *HSVTK*-MSC exosomes we report internalize both cell types and act intracellularly, thus overcoming drug resistance. This is supported by the observation that the *HSVTK* suicide gene delivered by lentiviral pseudotyped vectors mediated a complete tumor remission [24].

MSC-suicide gene exosomes provide several key advantages over intravenous administration of cells. Exosomes target tumor cells by means of internalizing them, thus bringing their cargo into cells. Therapy starts from within the cancer cell. Exosomes are not immunogenic, they easily penetrate the blood–brain barrier (BBB) and have higher safety standards over cells. Enhancement of anti-PD-1 immunotherapy in mouse models of glioblastoma was observed when tumor-targeting nanoparticles crossed the BBB [25]. Suicide gene therapies are often compared to the mythological Trojan horse concept in both cell-driven gene therapy and even more so when MSC-exosomes are involved in the treatment [14]. Several years ago, Trojan horse gene therapy approaches were excellently reviewed from different aspects [26]. Exosomes are natural extracellular vesicles that transfer messages between cells and act as cell’s waste eliminators. Combined with the tumor cell targeting ability of MSC, they are likely to be an ideal extracellular vesicle for cancer gene therapy in general. The tumor specific targeting of MSC-suicide gene exosomes is supported by our observations [3,4,5,7], where we used suicide gene transduced MSCs (producing exosomes) to treat tumor bearing rats or mice. We observed a therapeutic effect, but never any side effects. Recently, we reported experiments showing that neither human skin fibroblasts nor MSCs were susceptible to suicide gene yCD::UPRT-MSC exosomes [14]. Furthermore, MSC exosomes possessing classical cancer chemotherapeutic drugs in their cargo [27] are tumor cell targeted, thus supporting the tumor cell tropisms of MSC exosomes in general. Numerous recent research activities focusing on the engineering of artificial cancer-targeted nanoparticles are facing limitations when compared to MSC exosomes, mainly because of the toxic properties of the nanoparticle coat. The success of any given gene therapy is highly dependent on carrier efficiency. AT-MSCs modified with a lenti vector carrying *HSVTK* genes, injected at the tumor site, in the treatment of established intracranial glioma has recently been reported [28]. Like with *yCD::UPRT* CM, we have found that some *HSVTK* CM of naϊve MSCs showed stimulating tumor cell growth activity depending on tissue origin of MSC and on types of recipient tumor cells. Dental pulp mesenchymal stem/stromal cells labeled with iron sucrose release exosomes applied intra-nasally migrate to intracerebral glioblastoma [29]. Intranasal application of both *yCD::UPRT* [14] and *HSVTK* suicide gene exosomes may become a minimally invasive treatment method for brain tumors. MSC suicide gene exosomes may be worth testing in the treatment of diffuse intrinsic pontine glioma (DIPG). Patient-derived MSCs stably transduced with *HSVTK* as a source of individualized therapeutic exosomes could fulfill personalized treatment recommendations for DIPG [30]. From a practical point of view, the production of exosomes for potential clinical use does not require the same degree of skill and expenses as that required in cell handling. High stability of MSC-suicide gene exosomes and their intracellular activity allied them into a class of extracellular vesicles that can be stored on the shelf and injected intravenously. Suicide gene MSC exosomes carrying either *yCD::UPRT* or *HSVTK* genes bring new hope to patients with cancers that are difficult to treat. However, despite promising outcomes in vitro, running preclinical animal studies may shed more light on these expectations.

## 4. Materials and Methods

### 4.1. Culture Isolation and Maintenance of MSCs

All donors of adipose tissue, bone marrow, dental pulp, umbilical cord, blood platelets, and other tissue specimens used for isolation and propagation of MSCs were informed about the nature of the study and provided their written informed consent. All experimental protocols involving cells of human subjects were approved by the Ethical committee of the St. Elisabeth Cancer Institute (č. 4—2019/EK OUSA). We have previously described all MSC isolation procedures. AT-MSCs were isolated by collagenase digestion [3], bone marrow derived MSCs (BM-MSCs) were isolated using density gradient centrifugation [31], and dental pulp derived MSCs (DP-MSCs) and umbilical cord derived MSCs (UC-MSCs) were isolated from tissue fragments adhered to plastic tissue culture dishes [32]. For the expansion of all types of MSCs, the cells were seeded at 4000 cells/cm^2^ in plastic dishes (Corning Life Sciences, NY 14831 USA) and grown with medium exchange every 2–3 days. Adherent cells were split after reaching confluence with 0.05% trypsin/EDTA (Gibco, Hilden, Germany). All MSCs and *HSVTK*-MSCs used for the experiments were up to fifth passage or less. The number of exosomes per mL (24 h CM harvest) in such conditions was stable.

MSC cultures were grown as described previously [3]. Briefly, MSCs were cultivated in low glucose DMEM supplemented with 5% human platelet extract (PE) at 37 °C in a humidified atmosphere with 5% CO_2_. The tumor cell lines and primary glioblastoma cells were maintained in high glucose DMEM with 5% of fetal bovine serum. Human tumor cell lines used in this study were authenticated using STR profiling in 2018. All experiments were performed with mycoplasma-free cells.

### 4.2. Preparation of HSVTK-Retrovirus-Producing Cells

Recombinant retrovirus possessing the *HSVTK* gene was prepared as described previously [33]. Briefly, GP+E-86 helper cells were transfected with plasmid designated pAPtk containing the bicistronic retrovirus construct with the *HSVTK* gene separated by IRES sequence from the neo gene [12]. Virus-containing medium from G418-resistant ecotropic helper cells was used to infect amphotropic GP+envAM12 helper cells. Three to five ping-pong rounds of repeated infections were performed to obtain cells producing recombinant retrovirus particles with mixed envelope glycoproteins as described [12].

### 4.3. Cell Transduction with Retrovirus

All types of MSCs transduced with the *HSVTK*-gene by means of retrovirus infection were performed as previously described [11]. Briefly, subconfluent MSC cultures were infected three times in three consecutive days with a virus-containing medium supplemented with 100 μg/mL protamine sulfate. Cells were cultivated in selection media containing a pretested concentration of G418 able to kill naϊve cells (over a range of 0.4 to 1.2 mg/mL) for several days and expanded to obtain a homogenous population of *HSVTK*-gene transduced MSCs.

### 4.4. Cell Growth Assessment Using IncuCyte Live Cell Monitoring System

Cell growth was monitored by “real time in vitro micro-imaging” using the IncuCyte ZOOM™ Kinetic Imaging system (Essen BioScience, Hertfordshire SG8 5H, UK) and fluorescent microscopy (Axiovert 200, Zeiss, Jena, Germany). The IncuCyte system allows for the hourly monitoring of cell growth by determining the confluence of the cells and displaying the morphologic changes associated with treatment. The usual number of tumor cells plated into the wells of a 96-well plate was 3 × 10^4^. Most cell viability data obtained from the IncuCyte system were in good accordance with the data obtained by subjecting the plates to the CellTiter 96 Aqueous One Solution Cell Proliferation Assay MTT (Promega Corporation, Madison, WI 53711 USA).

### 4.5. Exosomes Isolation

Conditioned medium (CM) was the source of the exosomes. CM was collected from 90% confluent cell cultures with 90–97% cell viability assessed by Trypan-Blue exclusion (Sigma–Aldrich, St. Louis, MO, USA). The cells were washed with phosphate buffered saline (PBS) to remove any debris and cultured over 24 h in platelet extract (PE) deficient medium. The culture CM was centrifuged at 800 g for 5 min to remove cell debris and filtered through 0.22-μm syringe filter. Exosomes were isolated by size-exclusion chromatography on a Sepharose CL-2B column or by means of the exoEasy Maxi Kit (Qiagen, Hilden, Germany).

### 4.6. Polymerase Chain Reaction (PCR), RT-PCR, and qRT-PCR Analysis

Total DNAs were extracted from all types of HSVTK gene transduced MSCs and from BM-MSCs (as negative control) using the Genomic DNA from Tissue Purification Kit (Macherey-Nagel, Bethlehem, Pennsylvania, USA). Standard PCR was performed in 10 µL 2× Thermo Scientific PCR Master Mix, 0.5 µL of each primer for the *HSVTK* (10 µM) or *GAPDH* (10 µM) gene, 0.5 µL DNAs (20 ng/µL), and 3.5 µL nuclease-free water. Thermal cycling conditions for 35 cycles were initial denaturation at 95 °C for 2 min, denaturation at 95 °C for 15 s, annealing at 62 °C for 30 s, extension at 72 °C for 30 s, and final extension at 72 °C for 5 min.

For the endogenous control of amplification, the *GAPDH* gene was used. PCR with primers for *GAPDH* was performed with the same DNA, using the same thermal cycling conditions, except that the annealing temperature was 52 °C for the *GAPDH* primers.

The following primers were used:

5′ GGAGGACAACACATCGACCG 3′ (forward) and
5′ GCAGATACCGCACCGTATTGGC 3′ (reverse) for *HSVTK* (122 bp),
5′ CCACTCCTCCACCTTTGAC 3′ (forward) and
5′ ACCCTGTTGCTGTAGCCA 3′ (reverse) for *GAPDH* DNA (206 bp), cDNA (102 bp),
5′ TAGTCAAACCGGCACGATGTC 3′ (forward) and
5′ ATCAAGCAGTCGGAGCAGTTC 3′ (reverse) for *DSP* (80 bp).

PCR products were visualized by agarose gel electrophoresis (2%) and identified using a GeneRuler 50 bp DNA Ladder (Thermo Fisher Scientific, Waltham (HQ), MA, USA) and 1 kb DNA ladder Solis.

Total RNAs were isolated from all types of *HSVTK* gene transduced MSCs and from BM-MSCs (as negative control) using a RNeasy Mini Kit (Qiagen, Hilden, Germany). Reverse transcriptase reactions were performed using a RevertAid First Strand cDNA Synthesis Kit (Thermo Scientific). The presence of *HSVTK* in cDNA was confirmed by PCR with the same primers and conditions as for DNA. *GAPDH* was used as endogenous control gene of expression and amplification of cDNA. Using the same primers for *GAPDH* gene and same conditions as for DNA, PCR was performed with the same cDNA, and a fragment with 102 bp was obtained.

Real-time PCRs for DNA as well as for cDNA were carried out with Eco Real-Time PCR System Real-Time PCR System (Illumina, San Diego, CA 92122, USA) by the relative quantification method with primers for the *HSVTK* gene, *GAPDH* (housekeeping gene), or *DSP* (single copy gene) using a Maxima SYBR Green/ROX qPCR Master Mix (Thermo Scientific, Waltham (HQ), MA, USA), according to the manufacturer’s instructions. The amplification efficiencies of target amplification and reference (endogenous) amplification were approximately equal, determined by standard curve experiments. Results were analyzed by Eco Software v5.0 (Illumina). Gene expression and gene copy number were compared using the delta cycle threshold (ΔCt = Ct_TK_ − Ct_reference gene_) values for the respective cells examined where *GAPDH* expression or *DSP* gene amount was taken as an endogenous reference. Analysis was performed in triplicate and data expressed as mean ± SD. *HSVTK*-MSCs were taken as a reference sample. Change in gene expression was calculated according to the formula $RQ=2^{-∆∆Ct}$.

### 4.7. Isolation and Detection of mRNA from HSVTK-MSC Exosomes

*HSVTK*-MSC exosomes were isolated by means of the exoEasy Maxi Kit (Qiagen, Hilden, Germany) from CM. The exoRNeasy Serum/Plasma Starter Kit (Qiagen, Hilden, Germany) was used to isolate total RNA. Synthesis of cDNA from the exosome’s RNA was performed with a Thermo Scientific™ RevertAid™ First Strand cDNA Synthesis Kit (Thermo Scientific) using either random hexamer or oligo (dT)_18_ primers and analyzed by PCR using primers for *HSVTK* and *GAPDH* amplification.

### 4.8. Statistical Analysis

Unless noted otherwise, all experiments were repeated at least three times to enable statistical analysis and all results were similar between replicates. Error bars represent the standard error of the mean from independent samples within each experiment. All statistical analyses were performed using GraphPad Prism 4 software.

## 5. Conclusions

MSCs engineered to express herpes simplex virus thymidine kinase (*HSVTK*) secrete exosomes possessing mRNA of the *HSVTK* gene in their cargo. *HSVTK*-MSC exosomes internalized specific tumor cells and in the presence of prodrug ganciclovir (GCV) kill recipient tumor cells by intracellular conversion of ganciclovir to GCV triphosphate. *HSVTK*-AT-MSCs exosomes in GCV presence effectively inhibit human glioma cell lines and primary glioblastoma cells. *HSVTK*-MSC exosomes are cancer cell-specific drugs acting inside the tumor cell, thus overcoming resistance and side effects.

## References

[1] Studeny M., Marini F.C., Champlin R.E., Zompetta C., Fidler I.J., Andreeff M. (2002). Bone marrow derived mesenchymal stem cells as vehicles for interferon-beta delivery into tumors. Cancer Res..

[2] Altaner C. (2008). Prodrug Cancer Gene Therapy, Review. Cancer Lett..

[3] Kucerova L., Altanerova V., Matuskova M., Tyciakova S., Altaner C. (2007). Adipose tissue-derived human mesenchymal stem cells mediated prodrug cancer gene therapy. Cancer Res..

[4] Kucerova L., Matuskova M., Pastorakova A., Tyciakova S., Jakubikova J., Bohovic R., Altanerova V., Altaner C. (2008). Cytosine deaminase expressing human mesenchymal stem cells mediated tumour regression in melanoma bearing mice. J. Gene Med..

[5] Cavarretta I.T., Altanerova V., Matuskova M., Kucerova L., Culig Z., Altaner C. (2010). Adipose tissue-derived mesenchymal stem cells expressing prodrug-converting enzyme inhibit human prostate tumor growth. Mol. Ther..

[6] Abrate A., Buono R., Canu T., Esposito A., Del Maschio A., Lucianò R., Bettiga A., Colciago G., Guazzoni G., Benigni F. (2014). Mesenchymal stem cells expressing therapeutic genes induce autochthonous prostate tumour regression. Eur. J. Cancer.

[7] Altanerova V., Cihova M., Babic M., Rychly B., Ondicova K., Mravec B., Altaner C. (2012). Human adipose tissue-derived mesenchymal stem cells expressing yeast cytosinedeaminase::uracil phosphoribosyltransferase inhibit intracerebral rat glioblastoma. Int. J. Cancer.

[8] Altaner C., Altanerova V., Cihova M., Ondicova K., Rychly B., Baciak L., Mravec B. (2014). Complete regression of glioblastoma by mesenchymal stem cells mediated prodrug gene therapy simulating clinical therapeutic scenario. Int. J. Cancer.

[9] Altaner C., Shah K. (2013). Stem Cell-Mediated Prodrug Gene Therapy of High-Grade Brain Tumors, In Stem Cell Therapeutics for Cancer.

[10] Moolten F.L. (1986). Tumor chemosensitivity conferred by inserted herpes thymidine kinase genes: Paradigm for a prospective cancer control strategy. Cancer Res..

[11] Matuskova M., Hlubinova K., Pastorakova A., Hunakova L., Altanerova V., Altaner C., Kucerova L. (2010). HSV-tk expressing mesenchymal stem cells exert bystander effect on human glioblastoma cells. Cancer Lett..

[12] Altaner C., Altanerova U., Duzgunes N. (2019). Mesenchymal Stem Cell Exosome-Mediated Prodrug Gene Therapy for Cancer. Suicide Gene Therapy: Methods and Protocols, Methods in Molecular Biology.

[13] Kanada M., Kim B.D., Hardy J.W., Ronald J.A., Bachmann M.H., Bernard M.P., Perez G.I., Zarea A.A., Ge T.J., Withrow A. (2019). Microvesicle-mediated delivery of minicircle DNA results in effective gene-directed enzyme prodrug cancer therapy. Mol. Cancer Ther..

[14] Altanerova U., Jakubechova J., Benejova K., Priscakova P., Pesta M., Pitule P., Topolcan O., Kausitz J., Zduriencikova M., Repiska V. (2019). Prodrug suicide gene therapy for cancer targeted intracellular by mesenchymal stem cell exosomes. Int. J. Cancer.

[15] Zhang H., Freitas D., Kim H.S., Fabijanic K., Li Z., Chen H., Mark M.T., Molina H., Martin A.B., Bojmar L. (2018). Identification of distinct nanoparticles and subsets of extracellular vesicles by asymmetric flow field-flow fractionation. Nat. Cell Biol..

[16] Freeman S.M., Abboud C.N., Whartenby K.A., Packman C.H., Koeplin D.S., Moolten F.L. (1993). Abraham, G.N. The “bystander effect”: Tumor regression when a fraction of the tumor mass is genetically modified. Cancer Res..

[17] Kruse C.A., Roper M.D., Kleinschmidt-DeMasters B.K., Banuelos S.J., Smiley W.R., Robbins J.M., Burrows F.J. (1997). Purified herpes simplex thymidine kinase retrovector particles. I. In vitro characterization, in situ transduction efficiency, and histopathological analyses of gene therapy-treated brain tumors. Cancer Gene Ther..

[18] Rainov N.G. (2000). A phase III clinical evaluation of herpes simplex virus type 1 thymidine kinase and ganciclovir gene therapy as an adjuvant to surgical resection and radiation in adults with previously untreated glioblastoma multiforme. Hum. Gene Ther..

[19] Elshami A.A., Saavedra A., Zhang H., Kucharczuk J.C., Spray D.C., Fishman G.I., Amin K.M., Kaiser L.R., Albelda S.M. (1996). Gap junctions play a role in the ’bystander effect’ of the herpes simplex virus thymidine kinase/ganciclovir system in vitro. Gene Ther..

[20] Miletic H., Fischer Y., Litwak S., Giroglou T., Waerzeggers Y., Winkeler A., Li H., Himmelreich U., Lange C., Stenzel W. (2007). Bystander killing of malignant glioma by bone marrow-derived tumor-infiltrating progenitor cells expressing a suicide gene. Mol. Ther..

[21] Cottin S., Gould P.V., Cantin L., Caruso M. (2011). Gap junctions in human glioblastomas: Implications for suicide gene therapy. Cancer Gene Ther..

[22] Singh S.K., Hawkins C., Clarke I.D., Squire J., Bayani J., Hide T., Henkelman R.M., Cusimano M.D., Dirks P.B. (2004). Identification of human brain tumour initiating cells. Nature.

[23] Hu W., Liu W. (2010). Side populations of glioblastoma cells are less sensitive to HSV-TK/GCV suicide gene therapy system than the non-side population. Vitro Cell Dev. Biol. Anim..

[24] Huszthy P.C., Giroglou T., Tsinkalovsky O., Euskirchen P., Skaftnesmo K.O., Bjerkvig R., von Laer D., Miletic H. (2009). Remission of invasive, cancer stem-like glioblastoma xenografts using lentiviral vector-mediated suicide gene therapy. PLoS ONE.

[25] Kim S.S., Harford J.B., Moghe M., Slaughter T., Doherty C., Chang E.H. (2019). A tumor-targeting nanomedicine carrying the p53 gene crosses the blood-brain barrier and enhances anti-PD-1 immunotherapy in mouse models of glioblastoma. Int. J. Cancer.

[26] Collet G., Grillon C., Nadim M., Kieda C. (2013). Trojan horse at cellular level for tumor gene therapies. Gene.

[27] Buddhadev Layek B., Sadhukha T., Panyam J., Prabh S. (2018). Nano-engineered mesenchymal stem cells increase therapeutic efficacy of anticancer drug through true active tumor targeting. Mol. Cancer Ther..

[28] Tamura R., Miyoshi H., Yoshida K., Okano H., Toda M. (2019). Recent progress in the research of suicide gene therapy for malignant glioma. Neurosurg. Rev..

[29] Altanerova U., Benejova K., Altanerova V., Tyciakova S., Rychly B., Szomolanyi P., Ciampor F., Cihova M., Repiska V., Ondicova K. (2016). Dental pulp mesenchymal stem/stromal cells labeled with iron sucrose release exosomes and cells applied intra-nasally migrate to intracerebral glioblastoma. Neoplasma.

[30] Mueller S., Jain P., Liang W.S., Kilburn C., Kline L., Gupta N., Panditharatna E., Magge S.N., Zhang B., Zhu Y. (2019). A pilot precision medicine trial for children with diffuse intrinsic pontine glioma-PNOC003: A report from the Pacific Pediatric Neuro-Oncology Consortium. Int. J. Cancer.

[31] Altaner C., Altanerova V., Cihova M., Hunakova L., Kaiserova K., Klepanec A., Vulev I., Madaric J. (2013). Characterization of mesenchymal stem cells of “no-options” patients with critical limb ischemia treated by autologous bone marrow mononuclear cells. PLoS ONE.

[32] Stanko P., Kaiserova K., Altanerova V., Altaner C. (2014). Comparison of human mesenchymal stem cells derived from dental pulp, bone marrow, adipose tissue, and umbilical cord tissue by gene expression. Biomed. Pap. Med. Fac. Univ. Palacky Olomouc. Czech Repub..

[33] Hlavaty J., Hlubinova K., Altaner C. (1999). Construction and testing of gene therapy retroviral vector expressing bacterial cytosine deaminase gene. Neoplasma.

